# COMPARISON BETWEEN RENDERING 3D-CT AND TRANSPARENT 3D-CT IN ACL TUNNEL POSITIONING

**DOI:** 10.1590/1413-785220172501167914

**Published:** 2017

**Authors:** MARCOS AMSTALDEN BARROS, TIAGO LAZZARETTI FERNANDES, DIMITRIS DIMITRIOU, ANDRÉ PEDRINELLI, ARNALDO JOSÉ HERNANDEZ

**Affiliations:** 1. Universidade de São Paulo, Faculdade de Medicina, Hospital das Clínicas, Instituto de Ortopedia e Traumatologia, São Paulo, SP, Brazil.; 2. Massachusetts General Hospital and Harvard Medical School, Department of Orthopedic Surgery, Bioengineering Laboratory, Boston, MA, USA.

**Keywords:** Anterior cruciate ligament reconstruction. Imaging, Three-Dimensional. Image processing, computer-assisted

## Abstract

**Objective:**

To compare the transparent 3D computed tomography (CT) image protocol against conventional 3D-CT image-rendering protocol to assess femoral tunnel position in anatomic anterior cruciate ligament (ACL) reconstructions***.***

**Methods:**

Eight knee CT scans from cadavers were analyzed by image rendering 3D-CT protocol, using Rhinoceros^(r)^ software. The central point of the ACL tunnel was set using the sagittal plane. Same CT scans were analyzed using transparent 3D-CT measurement protocol with OsiriX^(r)^ software. Central point of the ACL tunnel was set using sagittal, coronal and axial planes. The grid system described by Bernard and Hertel was used to compare tunnel positions between protocols, using height and length parameters***.***

**Results:**

There was a significant difference between measurements using image rendering 3D-CT and transparent 3D-CT protocol for height (23.8 ± 7.9mm and 33.0 ± 5.0mm, respectively; p=0.017) and no differences for length (18.6 ± 4.2mm and 18.3 ± 4.5mm, respectively; p=0.560)***.***

**Conclusion:**

Height in transparent CT protocol was different and length was the same as compared to 3D-CT rendering protocol in Bernard and Hertel method for tunnel measurements. ***Level of Evidence II, Descriptive Laboratory Study.***

## RESUMO

**Objetivo:**

***Comparar o protocolo de tomografia computadorizada (TC) 3D por transparência com o protocolo TC-3D por renderização de imagem na avaliação do posicionamento de túneis femorais na reconstrução anatômica do ligamento cruzado anterior (LCA) .***

**Método:**

***^_Oito TC de joelho de cadáveres foram analisadas pelo protocolo de renderização de imagem 3D-TC utilizando o software Rhinoceros(r). O ponto central do túnel do LCA foi definido pelo plano sagital. As mesmas tomografias foram analisadas pelo protocolo 3D-TC por transparência, com o software OsiriX(r). O ponto central do túnel do LCA foi definido pelos planos sagital, coronal e axial. O sistema de grade de Bernard e Hertel foi utilizado para comparar a posição dos túneis entre os protocolos, utilizando parâmetros para comprimento e altura._^***

**Resultados:**

***Houve diferença significativa entre as medidas dos protocolos de renderização de imagem 3D-TC e 3D-TC por transparência para altura (23,8 ± 7,9mm e 33,0 ± 5,0mm, respectivamente; p=0,017), sem diferenças para comprimento (18,6 ± 4,2mm e 18,3 ± 4,5mm, respectivamente; p=0,560) .***

**Conclusão:**

*A altura no protocolo de TC por transparência foi diferente e o comprimento foi igual quando comparados com o protocolo 3D-TC por renderização de imagem no método de Bernard e Hertel para mensuração dos túneis.* ***Nível de Evidência II, Estudo Laboratorial Descritivo.***

## INTRODUCTION

Recent research on anterior cruciate ligament (ACL) reconstruction has endorsed restoration of the original anatomy.^1^ ^,^ ^2^ Correct placement of the femoral and tibial tunnels may help restore physiological relationships and ensure near-normal function of the knee joint. Because graft positioning is the most important intraoperative variable for surgical success,^3^ accurate analysis of the tunnel position is especially relevant in terms of quality control and improvement in ACL reconstruction.

The quadrant method, originally described by Bernard et al.,^4^ is the most commonly used reference for location of the ACL, originally described for the lateral x-ray of the distal femur.^5^ ^-^ ^7^ Gold standard imaging technique for evaluating osseous anatomy of the knee and anatomical femoral tunnel position is the rendering 3D computed tomography (CT) scan.^8^ ^-^ ^15^ However, conventional 3D CT rendering is time consuming and technically demanding. A less demanding open-source 3D CT protocol based on the principles of Bernard and Hertel x-ray method has recently been introduced and suggested for ACL femoral tunnel measurement.^16^

Therefore, the aim of the present study was to compare the transparent CT image protocol against the conventional 3D CT image rendering protocol. The hypothesis was that these two different protocols would properly evaluate osseous landmarks and femoral tunnel positioning in anatomical ACL reconstruction.

## MATERIAL AND METHODS

We evaluated 8 unilateral knee CT scans of cadavers, with the approval of the University of São Paulo Medical School Institutional Review Board (CEP no 436/11). All CT scans were from male subjects with an average age of 63.2 +/- 10.6 years.

All subjects were scanned in supine position from the mid-pelvis to the proximal tibia following the same protocol, using a 64-slice multi-slice spiral CT scanner (LightSpeed Plus, GE Medical Systems, Milwaukee, WI) with 120kV and 80mA settings. The images were acquired along the axial direction with a 1.25 mm slice thickness, in-plane resolution of 0.74 0.74 mm, and matrix size of 512 x 512.

Outside-in anatomic ACL reconstruction technique was used. Tunnels were created by the same senior surgeon in each knee in the center of the original ACL, above the ACL footprint remnants. Tunnels were created but grafts were not positioned, since they were not necessary for the present study.

### 3D CT image rendering protocol

**Figure 1:**
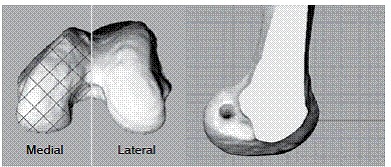
Rendered 3D CT protocol scan using Rhinoceros^(r)^ software. (A) Axial view, with most superior intercondylar notch plane set. (B) Lateral view after medial condyle was cropped, showing central point of ACL tunnel (green dot) at the medial wall of the lateral condyle.

Bone was rendered from the axial CT slices using Amira software (FEI Visualization Sciences Group, Bordeaux, France),^17,18^ processed with Geomagic Studio software (Research Triangle Park, NC) and analyzed with Rhinoceros software (McNeel North America, Seattle, WA). Lateral view was standardized by aligning posterior femoral condyle wall in the sagittal and axial planes and inferior wall in the sagittal and coronal views (in other words, following the protocol by Bird et al.).^9^ A coordinate system parallel to this view was created and dislocated to the most superior aspect of the intercondylar notch.^18^ (Figure 1A)

Medial femoral condyle using Rhinoceros^(r)^ tools according to the parallel plane created in the previous step. Central point of the ACL tunnel position was set using the sagittal plane. (Figure 1B)

### Transparent 3D CT measurement protocol

**Figure 2:**
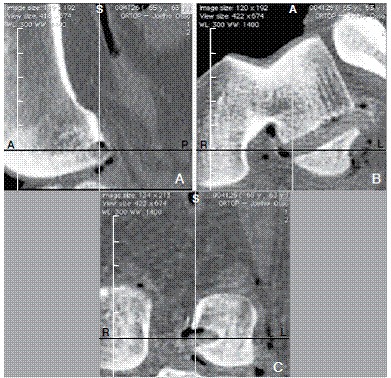
Sagittal (A), coronal (B), and axial (C) views of the central point of the ACL tunnel using OsiriX(r) Imaging Software.

Using an open-source software (OsiriX Imaging Software, http://www.osirix-viewer.com), 3D surface models of the femur were reconstructed from CT images (DICOM) using gradient threshold and region growing. Images were acquired including intercondylar notch and lateral condyle in a bone transparent imaging technique similar to radiographies (transparency 3D MPR protocol) available in this software.^16^ As mentioned before, posterior femoral condyle walls were aligned in sagittal and axial view and inferior walls in sagittal and coronal views to standardize the lateral view. Central point of the ACL tunnel position was set using sagittal, coronal and axial planes. (Figure 2)

### Bernard and Hertel method.

**Figure 3:**
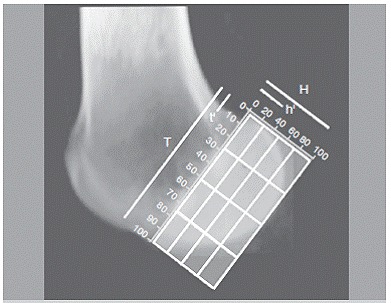
Bernard and Hertel^4^ quadrant method in a neutral transparent CT scan of the lateral femoral condyle. T = total condyle length, t'= central ACL percentage of T; H = total height, h' = central ACL percentage of H.

The grid system described by Bernard and Hertel^4^ was used to determine the tunnel position. In a lateral view, a line tangent to the roof of the intercondylar notch (Blumensaat's line) was drawn. Two lines were drawn perpendicular to this line, one at the intersection of the tangent line with the shallow border of the lateral femoral condyle and the other with intersection of the tangent line and the deep border of the lateral femoral condyle. Another line parallel to Blumensaat's line and tangent to the inferior border of the condyles was drawn to form the grid. ACL tunnel's central point was measured using height and length parameters. (Figure 3)

### Comparison between both protocols.

The same world coordinate system (WCS) was used for both methods. The central point of the femoral tunnel was projected from one system (OsiriX) to the other (Rhinoceros) onto the lateral condyle. The same lateral view was used again, as described previously. 

Distance between the two points was measured utilizing height (H) and length (T) parameters by Bernard and Hertel's method in milimeters +/- standard deviation and absolute distance between tunnels was also measured.

### Statistics

We tested data for normality and variance. Because distribution was normal, we performed paired t-tests (P < 0.05) using SigmaPlot 12.5 for Windows software. We calculated the sample size and the power of the study starting with the primary outcome.

## RESULTS

Both of the compared parameters (length and height) passed the normality test (Shapiro-Wilk); Two-tailed P-value for length was 0.560, and for height was 0.017.

Mean values for lenght (T) measurements using Rhinoceros and OsiriX were 18.6 +/- 4.2 and 18.3 +/- 4.5, respectively (P = 0.560). 

The mean values for height (H) measurements using Rhinoceros and OsiriX were 23.8 +/- 7.9 and 33.0 +/- 5.0, respectively (P = 0.017).

**Figure 4:**
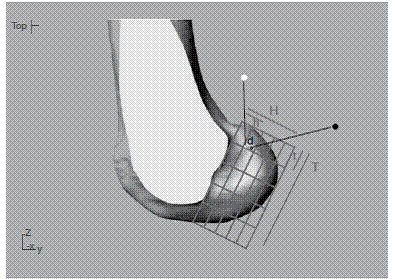
Lateral view of the medial femoral condyle. Red dot indicates mean center of ACL tunnel position using Rhinoceros. Blue dot represents the same position using OsiriX. Note the difference in height (H) between radiological methods. Distance between tunnels (d) = 2.02 mm.

Figure 4 shows mean localization of ACL tunnel's central point according to both methods (Rhinoceros and OsiriX) and also the distance between the central tunnel position using both methods.

**Figure 5:**
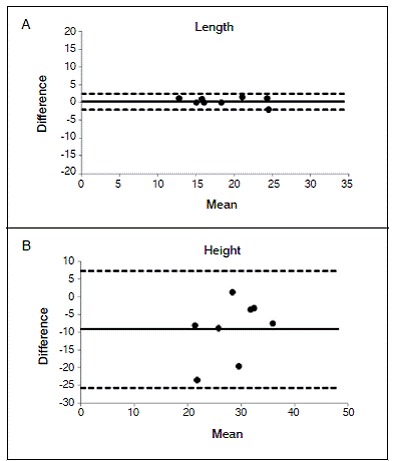
Bland-Altman plots analyzing the agreement of both methods for measuring length (A) and height (B) of femoral ACL tunnels. Y axis shows the difference between the two paired measurements and X axis represents the average of these measures. Solid line represents mean measurement values using both methods and dotted lines represent limits of agreement, from -1.96s to +1.96s. Note high correlation between methods for measuring length, but not for height.

Comparison of both methods found a significant difference between groups for height, and no differences for length. (Figure 5)

## DISCUSSION

Precise studies of tunnel positions in knees with ACL reconstruction can prevent inaccurate positioning and consequent negative outcomes. Non-anatomical graft placement is one of the most common causes of failure in ACL reconstruction. Marchant et al. found a misplaced femoral or tibial graft tunnel in 107 of 122 (88%) patients with failed ACL reconstructions.^1^ The kinematics of the reconstructed knee are altered by the position of the femoral and tibial tunnels. Anatomical ACL reconstruction restores original stability closer to the native ACL and provides better knee kinematics when compared to non-anatomical ACL reconstruction.^19^

The clinical relevance of the present study is intimately related to the importance of correct reporting of tunnel positioning to compare post-surgical outcomes in ACL anatomic reconstruction. Van Eck et al.^20^ states that outcomes should be reported and compared in a similar and thorough manner for valid interpretation. Post-operative CT investigation of ACL reconstruction has the potential to improve surgical technique and the novel transparent 3D CT imaging protocol can contribute to this outcome.

The present study compared two methods for analyzing post-operative femoral tunnel position in ACL reconstruction: the novel transparent 3D CT image protocol and the conventional 3D CT image rendering protocol. This comparison produced similar results for length measurements but different results for height. Consequently, different ACL reconstruction procedures can be compared by properly choosing one of the imaging evaluation methods separately. Surgeons can choose one of the methods to evaluate femoral tunnel positioning in anatomic ACL reconstruction, but they should not compare the methods.

The main advantage of the conventional 3D CT image-rendering protocol is that it is already established and accepted as the preferred method for evaluating femoral ACL tunnel positioning whenever precise measurements are needed.^10^ The disadvantage of this method is that it requires specific and consequently more onerous training to correctly assess and interpret data, as well as more expensive software for image processing. 

The transparent 3D CT protocol has already been proven to accurately measure the ACL femoral tunnel,^16^ and uses an open-source software that requires a lower level of technical skill. However, this tool is chosen less frequently when compared to the conventional 3D CT image-rendering protocol.

Limitations of the present study include assessing only femoral tunnel position but not tibial. Although it is the position of the femoral tunnel that plays a major role in providing graft isometricity, it would be interesting to analyze the tibial tunnel position in future studies; questions related to the measurement of tunnel positions in the coronal and axial planes might also emerge. Measurements were taken only in the lateral view since this is where ACL tunnels are positioned. Furthermore, the tunnel angulation in the coronal and axial planes does not alter knee kinematics to the same degree as variations in the sagittal plane. Finally, height and length parameters were measured but the center angle of the ACL graft was not analyzed. Future studies are proposed to study these parameters, as well as variations with internal and external femoral axis rotation or adduction and abduction of the cropped medial femoral condyle in the rendered CT protocol. These variations should make a difference in measurement outcomes.

## CONCLUSION

Height in the transparent 3D CT image protocol was different and length was equal when compared to the 3D CT image-rendering protocol using the Bernard and Hertel method for tunnel measurement.

## References

[1] Marchant BG, Noyes FR, Barber-Westin SD, Fleckenstein C (2010). Prevalence of nonanatomical graft placement in a series of failed anterior cruciate ligament reconstructions. Am J Sports Med.

[2] Aglietti P, Buzzi R, Giron F, Simeone AJ, Zaccherotti G (1997). Arthroscopic-assisted anterior cruciate ligament reconstruction with the central third patellar tendon. A 5-8-year follow-up. Knee Surg Sports Traumatol Arthrosc.

[3] Topliss C, Webb J (2001). An audit of tunnel position inanterior cruciate ligament reconstruction. Knee.

[4] Bernard M, Hertel P, Hornung H, Cierpinski T (1997). Femoral insertion of the ACL. Radiographic quadrant method. Am J Knee Surg.

[5] Colombet P, Robinson J, Christel P, Franceschi JP, Djian P, Bellier G (2006). Morphology of anterior cruciate ligament attachments for anatomic reconstruction: a cadaveric dissection and radiographic study. Arthroscopy.

[6] Takahashi M, Doi M, Abe M, Suzuki D, Nagano A (2006). Anatomical study of the femoral and tibial insertions of the anteromedial and posterolateral bundles of human anterior cruciate ligament. Am J Sports Med.

[7] Zantop T, Wellmann M, Fu FH, Petersen W (2008). Tunnel positioning of anteromedial and posterolateral bundles in anatomic anterior cruciate ligament reconstruction: anatomic and radiographic findings. Am J Sports Med.

[8] Basdekis G, Christel P, Anne F (2009). Validation of the position of the femoral tunnels in anatomic double-bundle ACL reconstruction with 3-D CT scan. Knee Surg Sports Traumatol Arthrosc.

[9] Bird JH, Carmont MR, Dhillon M, Smith N, Brown C, Thompson P (2011). Validation of a new technique to determine midbundle femoral tunnel position in anterior cruciate ligament reconstruction using 3-dimensional computed tomography analysis. Arthroscopy.

[10] Hoser C, Tecklenburg K, Kuenzel KH, Fink C (2005). Postoperative evaluation of femoral tunnel position in ACL reconstruction: plain radiography versus computed tomography. Knee Surg Sports Traumatol Arthrosc.

[11] Hoshino Y, Kim D, Fu FH (2012). Three-dimensional anatomic evaluation of the anterior cruciate ligament for planning reconstruction. Anat Res Int.

[12] Iwahashi T, Shino K, Nakata K, Otsubo H, Suzuki T, Amano H (2010). Direct anterior cruciate ligament insertion to the femur assessed by histology and 3-dimensional volume-rendered computed tomography. Arthroscopy.

[13] Kopf S, Forsythe B, Wong AK, Tashman S, Anderst W, Irrgang JJ (2010). Nonanatomic tunnel position in traditional transtibial single-bundle anterior cruciate ligament reconstruction evaluated by three-dimensional computed tomography. J Bone Joint Surg Am.

[14] Kopf S, Musahl V, Tashman S, Szczodry M, Shen W, Fu FH (2009). A systematic review of the femoral origin and tibial insertion morphology of the ACL. Knee Surg Sports Traumatol Arthrosc.

[15] Purnell ML, Larson AI, Clancy W (2008). Anterior cruciate ligament insertions on the tibia and femur and their relationships to critical bony landmarks using high-resolution volume-rendering computed tomography. Am J Sports Med.

[16] Fernandes TL, Martins NM, Watai FA, Albuquerque C, Pedrinelli A, Hernandez AJ (2015). 3D computer tomography for measurement of femoral position in acl reconstruction. Acta Ortop Bras.

[17] Chen CH, Li JS, Hosseini A, Gadikota HR, Gill TJ, Li G (2012). Anteroposterior stability of the knee during the stance phase of gait after anterior cruciate ligament deficiency. Gait Posture.

[18] Forsythe B, Kopf S, Wong AK, Martins CA, Anderst W, Tashman S (2010). The location of femoral and tibial tunnels in anatomic double-bundle anterior cruciate ligament reconstruction analyzed by three-dimensional computed tomography models. J Bone Joint Surg Am.

[19] Hong L, Li X, Zhang H, Liu X, Zhang J, Shen JW (2012). Anterior cruciate ligament reconstruction with remnant preservation: a prospective, randomized controlled study. Am J Sports Med.

[20] van Eck CF, Schreiber VM, Mejia HA, Samuelsson K, van Dijk CN, Karlsson J (2010). "Anatomic" anterior cruciate ligament reconstruction: a systematic review of surgical techniques and reporting of surgical data. Arthroscopy.

